# Clonal hematopoiesis in lung adenocarcinoma pathogenesis

**DOI:** 10.1172/jci.insight.203981

**Published:** 2026-05-08

**Authors:** Tiziana Parisi, Blanca Santibanez Ocampo, Jacob Adelman, Yuyan Cai, Marie McConkey, Christopher J. Gibson, Benjamin L. Ebert, Peter G. Miller, Tyler Jacks

**Affiliations:** 1 Koch Institute for Integrative Cancer Research, Massachusetts Institute of Technology, Cambridge, Massachusetts, USA.; 2 Dana Farber Cancer Institute, Boston, Massachusetts, USA.; 3 Broad Institute of MIT and Harvard, Cambridge, Massachusetts, USA.; 4 Krantz Family Center for Cancer Research Massachusetts General Hospital, Boston, Massachusetts, USA.

**Keywords:** Hematology, Oncology, Clonal selection, Hematopoietic stem cells, Lung cancer

## Abstract

<article data-scroll-anchor=”true” data-testid=”conversation-turn-2” data-turn=”assistant” data-turn-id=”request-WEB:b9841efa-619a-4cf3-8708-cec126c071b3-0” dir=”auto” tabindex=”-1”> Age-related blood cell mutations (clonal hematopoiesis) reshape immune cells and lung tumor immune structures, but did not increase lung cancer growth in a genetically engineered mouse model. </article>

Clonal hematopoiesis (CH) is an age-associated expansion of hematopoietic stem cells (HSC) clones harboring somatic mutations. Individuals with CH have higher mortality and increased risk of hematologic malignancies, cardiovascular disease, and inflammatory conditions, likely driven by aberrant inflammatory programs in mutant immune cells (1). For example, *TET2* loss leads to exaggerated IL-1β and IL-6 responses after LPS exposure.

CH is enriched in individuals with solid tumors and correlates with higher mortality. However, whether mutant hematopoietic populations causally enhance tumor growth remains unclear. The relationship may be multifactorial, reflecting prior cytotoxic exposure and the immunomodulatory effects of CH-derived myeloid cells. In patients with CH and non–small cell lung cancer (NSCLC), mutant hematopoietic cells within tumor biopsies were recently shown to associate with worse cancer outcomes. Using a mouse model of CH and orthotopic NSCLC implantation, the authors found that CH altered tumor immune composition but did not report in vivo tumor growth phenotypes (2).

To define the effect of hematopoietic *Tet2* loss on tumor development in vivo, we used congenic, autochthonous *TET2*-mutant CH mouse models combined with lung adenocarcinoma (LUAD) driven by inducible *KrasG12D* and *Trp53* loss (KP mice). Whole bone marrow from *Tet2^fl/fl^;Vav-Cre* (*Tet2* KO) or *Tet2^+/+^;Vav-Cre* (*Tet2* WT) donors was transplanted into lethally irradiated *Kras^LSL-G12D/+^;Trp53f^l/fl^* (KP) recipients (Figure 1A). *Tet2^fl/fl^;Vav-Cre* induces hematopoietic *Tet2* loss, while the KP system recapitulates LUAD development induced by intratracheal Adeno-Cre instillation (1, 3). Hematopoietic reconstitution was confirmed at 7 weeks, and tumors were induced with Adeno-Cre at 8 weeks.

Before tumor induction, *Tet2*-KO mice showed decreased peripheral B cells and increased T cells, monocytes, and neutrophils. By 16 weeks, only B cell differences remained significant (Figure 1B and Supplemental Figure 1A; supplemental material available online with this article; https://doi.org/10.1172/jci.insight.203981DS1). Splenic composition was also altered in tumor-bearing and non–tumor-bearing *Tet2*-KO mice, with more CD4^+^ and fewer CD8^+^ T cells and an increase in conventional DCs (cDCs, CD11c^+^MHCII^+^), skewed toward cDC2s over cDC1s (Supplemental Figure 1B).

Computed tomography and histopathology at 12 and 16 weeks showed no significant difference in tumor burden (tumor area per lung) or grade between *Tet2* WT and -KO mice (Figure 1C and Supplemental Figure 1C). Instead, *Tet2*-KO mice exhibited increased pulmonary lymphoid aggregates (LA) (Figure 1, D and E, and Supplemental Figure 1, D and E). These resembled tertiary lymphoid structures (TLS), immune aggregates that modulate antitumor responses, including in NSCLC (4). LA numbers per lung were significantly higher in *Tet2*-KO mice with and without tumors than in WT (approximately 2- to 3-fold).

IHC analyses revealed distinct LAs and tumor microenvironment (TME) composition by hematopoietic genotype. In the absence of tumors, LAs in *Tet2*-KO mice contained more CD4^+^ and fewer Foxp3^+^ T cells, as well as fewer Ly6G^+^ myeloid cells, while trends observed in B cells and macrophages became significant in tumor-bearing lungs (Figure 1E). Of note, however, within the TME, only differences in macrophages and CD8^+^ T cells were significant in *Tet2*-KO versus WT mice (Figure 1F).

Together, these data show that *Tet2*-mutant hematopoiesis reshapes immune cell composition and LA abundance in the blood, spleen, lung parenchyma, and LUAD TME. LA number and composition depended on hematopoietic genotype and tumor presence, consistent with their role in antitumor immunity and checkpoint inhibitor response in lung cancer. Despite these immune alterations, LUAD burden and grade were unchanged, suggesting that *Tet2*-mutant hematopoiesis modulates the immune landscape without directly promoting tumor growth.

Our findings align partly with prior reports using orthotopic tumor implantation models of *Tet2*-mutant CH. One study reported a myeloid-enriched TME without changes in tumor burden, though LAs were not evaluated (2). Another study using myeloid-specific *Tet2* deletion found increased tumor growth and more granulocytes in the TME but did not assess LAs (5). Differences in experimental design, timing, and tissue context likely explain these differences.

The KP model offers important advantages for studying LUAD initiation and progression in situ, but it also has limitations. Tumors driven by *Kras^G12D^* activation and *Trp53* loss are aggressive and relatively nonantigenic, which may obscure subtle immune effects and limit the assessment of CH influence on metastatic potential. Bone marrow transplantation used to establish CH may also alter T cell repertoire and immune tone. Finally, our experiments did not incorporate cytotoxic or immunotherapeutic exposures, which are clinically relevant modifiers of CH biology and might reveal additional interplay between mutant hematopoietic cells and tumor immunity, as recently shown with *TET2*-mutant CH (6).

In summary, *Tet2*-mutant CH reshapes systemic and tissue-level immune architecture, including expansion of tertiary lymphoid-like structures, but it does not accelerate autochthonous LUAD progression in KP mice. These findings show that CH can reshape the tumor immune microenvironment without accelerating tumorigenesis, supporting future studies across CH genotypes, tumor models, and therapeutic contexts.

## Author contributions

TP, PM, and TJ designed and conceived the study. TP, PM, CJG, JA, BSO, YC, and MM performed the experiments. TP, PM, JA, BSO, YC, TJ, and BLE analyzed and interpreted the data. PM and TP drafted the manuscript.

## Conflict of interest

In the past 3 years, PM has received consulting fees from Foundation Medicine Inc. TP is a current board member and holder of stock options in Paralog Therapeutics Inc. TJ is a member of the Board of Directors of Amgen and Thermo Fisher Scientific and is a cofounder of Dragonfly Therapeutics. TJ serves on the Scientific Advisory Board of Dragonfly Therapeutics and Skyhawk Therapeutics. TJ is also President of Break Through Cancer. His laboratory currently receives funding from the Lustgarten Foundation.

## Funding support

This work is the result of NIH funding, in whole or in part, and is subject to the NIH Public Access Policy. Through acceptance of this federal funding, the NIH has been given a right to make the work publicly available in PubMed Central.

- NIH (K08-CA263181 to PM).
- Edward P. Evans Foundation (PM.)
- Department of Defense (DOD) (CA220652 to PM).
- Janssen Pharmaceutical Company (TP, BCSO, JA, YC).
- Koch Institute Support (core) frant P30-CA014051 from the National Cancer Institute (TP).

## Supplementary Material

Supplemental data

Supporting data values

## Figures and Tables

**Figure 1:**
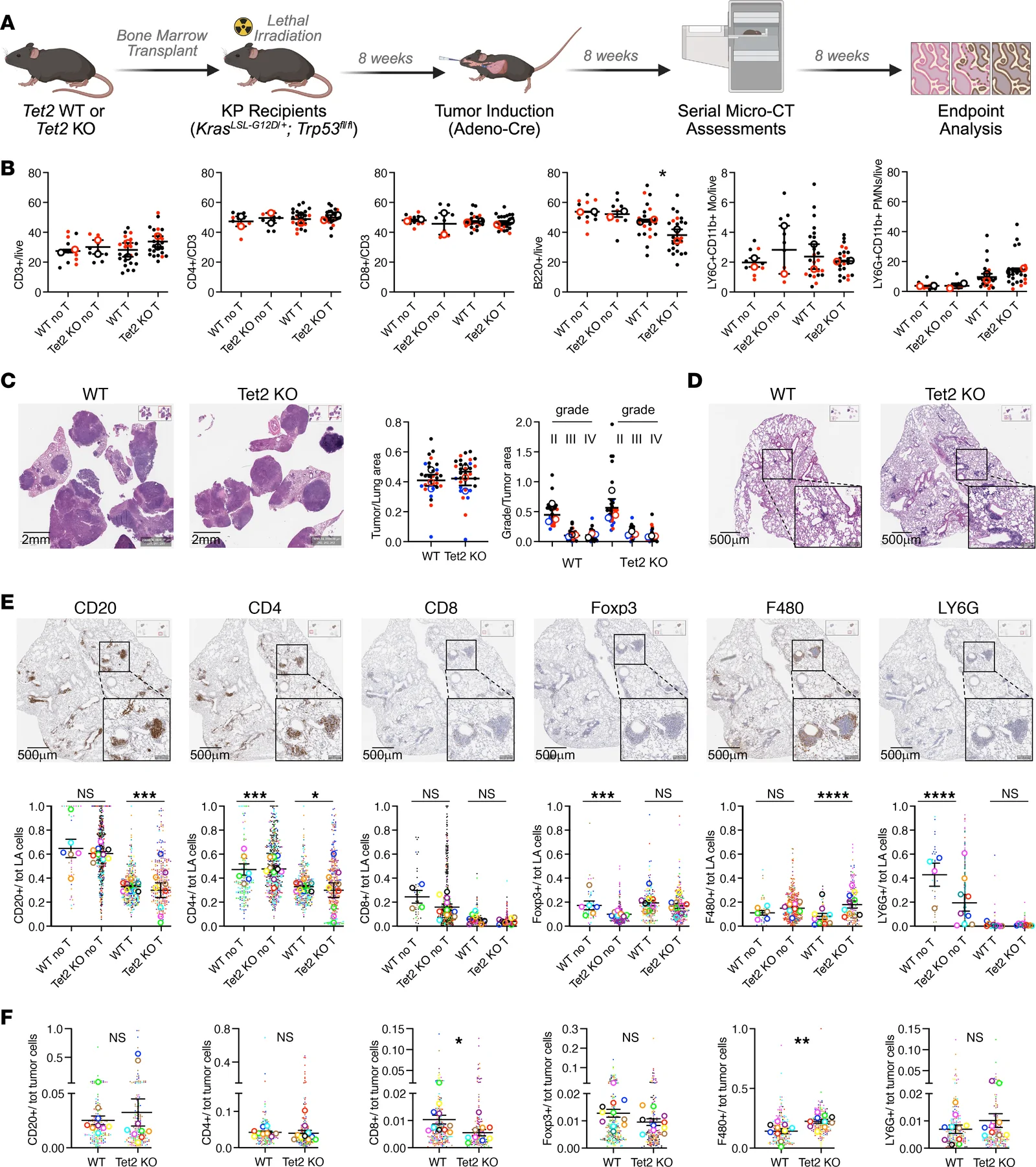
Effects of TET2-mutant CH on LUAD and immune cell repertoire. (**A**) Experimental approach. (**B**) Flow cytometry analyses of peripheral blood. Each dot represents a mouse (*n* = 11–24). (**C**) Tumor burden and grade at 16 weeks. Each dot represents a mouse (*n* = 30–32). (**D**) Images of lymphoid aggregates (LAs) in non-tumor bearing lungs. (**E**) IHC of lung from *Tet2* KO mouse (top) and relative quantification within LAs. Each color represents data from a single mouse (*n* = 4–11). (**F**) IHC quantification of immune cells within tumors. Each color represents data from a single mouse (*n* = 9–11). Data are from 2 or 3 independent experiments at 16-week time point (color-coded in **B** and **C**). Circles represent color-coded means, and data are shown as mean ± SEM. Statistical analyses were performed with 1-way ANOVA tests (**B**, Tukey, **E**, Brown-Forsythe and Welch nested), in **C** with the Mann-Whitney *U* test, and in **F** with 2-tailed nested *t* test of nested. T, tumor. *P < 0.05, **P < 0.01, ***P < 0.001, ****P < 0.0001.

## Version 1 05/08/2026

Electronic publication

## Version 2 09/03/2026

Peter G. Miller's name was updated
