# Supplementary material for: Clonal hematopoiesis in lung adenocarcinoma pathogenesis
Source: JCI Insight. 2026 May 8;11(9):e203981. doi: 10.1172/jci.insight.203981 (PMC13232000; doi:10.1172/jci.insight.203981)
Supplement: Supplemental data [file jciinsight-11-203981-s002.pdf]

SUPPLEMENTAL MATERIAL

Clonal Hematopoiesis in Lung Adenocarcinoma Pathogenesis

Authors: Tiziana Parisi, Blanca Santibanez Ocampo, Jacob Adelman, Yuyan Cai, Marie McConkey, Christopher J. Gibson, Benjamin L. Ebert, Peter G. Miller, Tyler E. Jacks

Conflict of Interest Statement

In the past three years, P.G.M. has received consulting fees from Foundation Medicine, Inc. Tiziana Parisi is a current board member and holder of stock options in Paralog Therapeutics Inc. T.E.J. is a member of the Board of Directors of Amgen and Thermo Fisher Scientific, and a co-Founder of Dragonfly Therapeutics. T.J. serves on the Scientific Advisory Board of Dragonfly Therapeutics and Skyhawk Therapeutics. T.J. is also President of Break Through Cancer. His laboratory currently receives funding from the Lustgarten Foundation, but these funds did not support the research described in this paper.

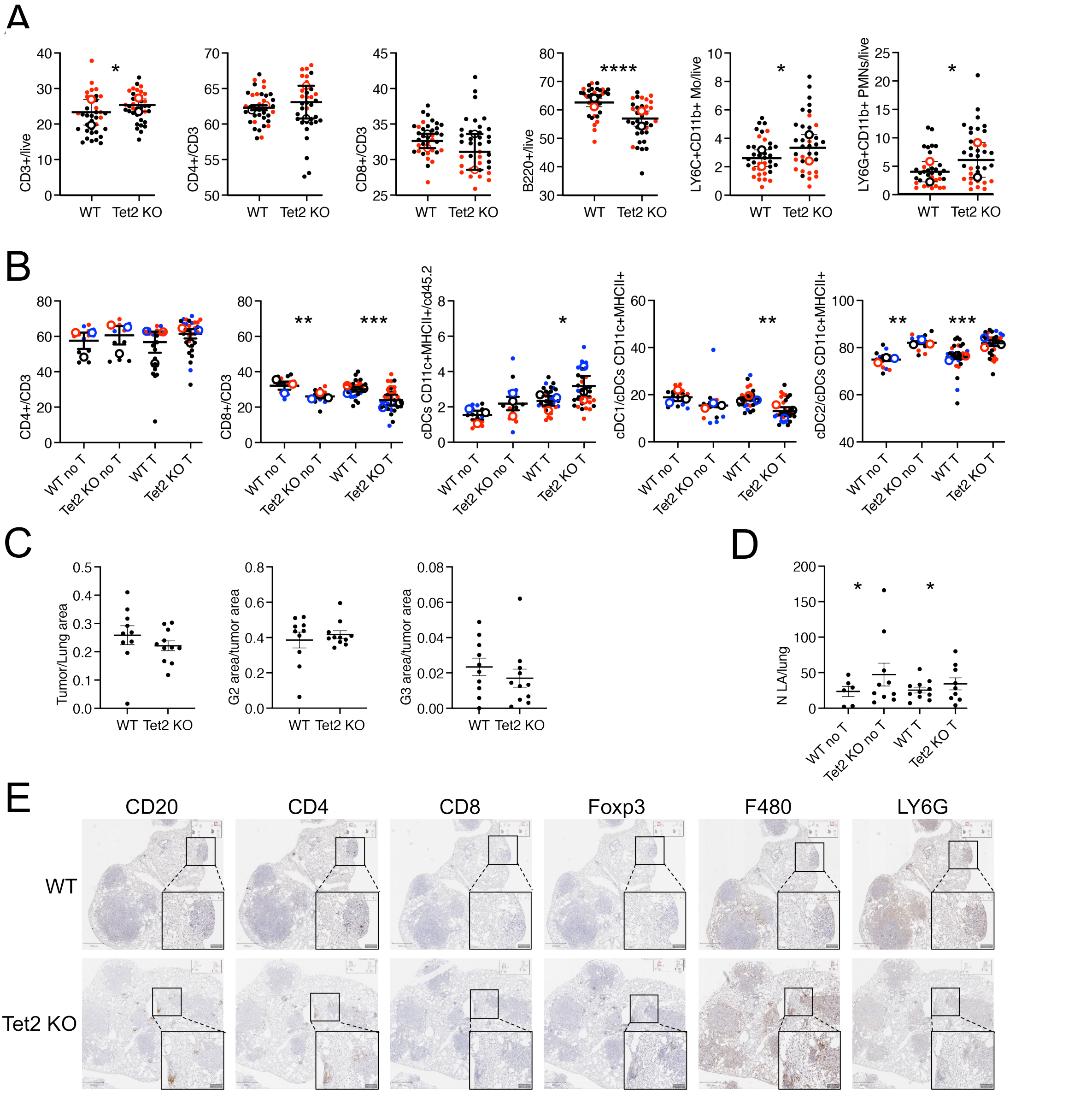

Supplemental Figure 1: Characterization of mouse model.

(A) Flow cytometry analyses of peripheral blood composition of Tet2 WT and KO mice at 7-weeks (one week prior to induction of tumors). Each dot represents a mouse (36-37 per group).
(B) Splenic immune cell composition assessed by flow cytometry at 16-week time point. Each dot represents a mouse 13-30 per group).
(C) Overall, Grade 3, and Grade 4 tumor burden at 12-week timepoint. Each dot represents a mouse (10-11 per group).
(D) Lymphoid aggregates per lung in healthy and tumor-bearing lungs. Each dot represents a mouse (6-10 per group).
(E) Representative images of lymphoid aggregates in tumor-bearing lungs.
Data shown in A are from two independent experiments, in B, D from three, and in C from one (color-coded in A and B). Circles represent averages, and error bars SEM. Statistical analyses were performed with the Mann-Whitney test (A, C and D), and one-way ANOVA with Tukey's multiple comparison tests (B). (T, tumor; WT, wild-type; KO, knockout. *p<0.05, **p<0.01, ***p<0.001, ****p<0.0001)

METHODS

Mouse models

To model clonal hematopoiesis, 4 × 10^6 whole bone marrow cells from CD45.2 *Tet2* wildtype (*$Tet2^{+/+}$;Vav-Cre^+*) or *Tet2* mutant (*$Tet2^{fl/fl}$;Vav-Cre^+*) mice were transplanted into lethally irradiated (500 Gy x 2 doses) recipient CD45.1 *$Kras^{LSL\text{-}G12D}$*; *$Trp53^{flox/flox}$* mice (6-10 weeks old). After 7 weeks, peripheral blood was collected by retro-orbital bleeding, and hematopoietic reconstitution confirmed by flow cytometric analysis through quantification of CD45.1 versus CD45.2 positive cells. To induce lung tumors, the reconstituted *$Kras^{LSL\text{-}G12D}$*; *$Trp53^{fl/fl}$* mice received intratracheal instillation of 2.5 × 10^8 plaque-forming units of adenovirus-expressing Cre driven by the alveolar type II cell-specific surfactant protein C promoter (SPC-Cre). All analyses were performed in approximately equal numbers of male and female mice. At endpoint animals were euthanized and dissected. Tissues were fixed in 4% paraformaldehyde or freshly used for flow cytometry analyses.

Tumor imaging and histological quantification

Lung tumor progression was monitored longitudinally by X-ray microcomputed tomography (µCT), and quantified at endpoint by histology. 5µm paraffin section were stained by H&E and analyzed by the investigators together with a pathologist. The slides were then scanned with an Aperio ScanScope AT2 at 20X magnification, and quantification of lung tumor burden and grade conducted by automated convolutional neural network (CNN)—developed in collaboration with Aiforia Technologies as previously described (Nat Genet. 2023 Sep 14;55(10):1686–1695. doi: 10.1038/s41588-023-01499-4). Aiforia algorithm v.NSCLC_v37_noheatmap was used. Tumor burden was calculated as total tumor area (sum of grades 1–4) divided by the total lung area. Tumor grade was calculated by dividing areas of each grade by the total tumor area.

Immunohistochemistry and quantification

For immunostaining deparaffinized sections were treated with 3% H_2O_2 for 15' followed by antigen retrieval (5′ at 125^0C in 10 mM sodium citrate pH 6.0, 0.05% Tween 20) and incubation with primary antibodies overnight at 4^0C. Detection and staining was performed with Vector ImmPRESS HRP and DAB kits (Vector laboratories). Slides were counterstained with hematoxylin. The area of positive and negative antibody staining was quantified by manual and/or automatic annotation in QuPath (Bankhead P, et al. QuPath: Open source software for digital pathology image analysis. Sci. Rep. 2017;7:16878. doi: 10.1038/s41598-017-17204-5. The following antibody were used for IHC in this study: CD8α (4SM16, eBiosciences), CD4 (EPR19514, Abcam), Foxp3 (FJK-16s, eBioscience), CD20 (SP32, Abcam), LY6G (1A8, BD), F4/80 (D2S9R, Cell Signaling)

Statistics

Statistical analyses were performed with GraphPad Prism v10 using the Mann-Whitney test (Figure 1C, Supplemental Figure 1A, C and D), two-tailed nested T test of nested (Figure 1F), one-way ANOVA with Tukey's multiple comparison tests (Figure 1B and Supplemental Figure 1B), or Brown-Forsythe and Welch ANOVA tests of nested (Figure 1E). Statistical significance is reported as *$P < 0.05$, **$P < 0.01$, ***$P < 0.005$, and ****$P < 0.001$.

Tissue preparation and staining for flow cytometry

Bone marrow was collected in PBS 3%FBS from femur, tibia and pelvis and filtered through a 100um cell strainer. The cells were spun at 300xg for 5', resuspended in ACK buffer for 5' to eliminate red blood cells (RBCs), and spun again at 300xg for 5'. The pellet was then resuspended in PBS 3%FBS, filtered through 40um cell strainer, and cells counted. Cells were spun at 300xg for 5', and resuspended in HBSS prior to retro-orbital injection into irradiated *$Kras^{LSL\text{-}G12D}$*; *$Trp53^{fl/fl}$* mice. To assess bone marrow reconstitution, peripheral blood was collected by retro-orbital bleeds into tubes with 100 µL of 4% sodium citrate, spun at 350xg for 5', and resuspended in 1 mL of ACK lysis buffer to eliminate RBCs. After 1' 5 mL of PBS 2% FBS (FACS buffer) were added. This was repeated a second time to remove excess RBCs.

To assess blood composition at the endpoint, peripheral blood was collected with a 1 ml syringe from the vena cava immediately after euthanasia, and processed as above. Spleens were dissociated using syringe backs on 70-micron filters, resuspended in RPMI 2% FBS, spun at 350G for 5', and treated with 2 ml ACK lysis buffer

for 2'. After quenching with FACS buffer, the mix was spun at 350xg for 5', resuspended in FACS buffer and processed for staining. Whole lungs or tumor-bearing lungs were collected, minced, and digested at 37 °C for 30 min with agitation in 3 ml of buffer containing 500 U/ml of collagenase type IV and 20 µg/ml of DNase (Sigma-Aldrich). RPMI 2%FBS were added post-incubation, and the homogenates passed through a 100-µm filter and spun at 350xg for 5'. The pellets were resuspended in 1 ml ACK lysis buffer, incubated for 2', and quenched with FACS buffer. Cells were spun at 350xg for 5', resuspended in FACS buffer, and processed for staining. To stain for flow cytometry, blood, spleen and lung cells were plated into 96-well U-bottom plates, and incubated on ice with FACS buffer+Fc block (1:200, Mo Fc CD16/CD32 block). The plates were spun for 5' at 1500 rpm, and incubated with a cocktail of directly conjugated antibodies recognizing surface proteins. After staining, cells were washed two times with 200 µL of FACS buffer and fixed with the BD CytoFix/Perm kit, or fixed with a Fixation/Permeabilization kit O/N at 4°C (eBioscience) for intracellular transcription factor (TF) staining. TF staining was performed for 1 h at RT in Permeabilization Buffer (eBioscience) and samples analyzed within few hours on a BD LSRFortessa four-laser flow cytometer . Data were analyzed in FlowJo v.10.10.0. Single cell suspensions were first gated on FSC-A versus SSC-A and then FSC-A versus FSC-H for singlets. For blood analyses, live cells (Zombie dye-negative), were either gated on CD45.1 and CD45.2 to assess the degree of chimerism, or on B220 versus CD3 to identify B (B220+) and T cells (CD3+) fractions. CD3+ T cells were further gated on CD4 versus CD8 positivity. The B220-CD3- population was gated on Ly6G and Cd11b to identify neutrophils (PMNs, Ly6G+Cd11b+), and the Ly6G-Cd11b+ fraction further analyzed for LY6C positivity to quantify monocytes (Ly6C+Cd11b+). Splenocyte suspensions were split to stain for myeloid cells or lymphocytes markers. For myeloid cells, live cells were gated on CD45.2, and the CD45.2+ cells analyzed for B220 versus CD3 positivity. The B220-CD3- population was then gated on Ly6G and Cd11b to identify neutrophils, and the Ly6G-Cd11b+ fraction further analyzed for double MHCII and Cd11c positivity. The dendritic cells (cDCs) so identified, were gated on CD172a and XCR1 to quantify the percentage of cDC1s (XCR1+CD172a-) and cDC2 (XCR1-CD172a+). The remaining Ly6G-Cd11b+Cd11c- fraction was finally analyzed for Ly6C positivity to identify monocytes. To characterize lymphocytes in the spleens, live cells were gated on CD3, and CD3+ T splenocytes further analyzed for CD4 versus CD8 positivity.

The Zombie Aqua™ Fixable Viability Kit was purchased from BioLegend (#423102), and the following anti-mouse antibodies were used for flow cytometry: **BD Biosciences:** Fc CD16/CD32 block, (#553142), BUV737 CD4 (#612843), BUV395 CD8α (#563786), BV421 Ly-6C (#562727), BUV395 F4/80 #565614. **BioLegend:** BV605 CD45.2 (#109841), BV605 CD45.1 (#110705), BV421 CD3 (#100227), BV785 CD11b (#101243), AF700 Ly-6G (#127622), PerCP/Cy5.5 CD45R/B220 (#103236), FITC CD172a (SIRPα) (#144006), PE XCR1 (#148204), APC CD64 (#139306). **eBiosciences:** APC-eFluor™ 780 MHC II (#47-5321-82), PE-Cy7 CD11c (#25-0114-82).

<u>Study Approval</u>
All animal use was approved by the Department of Comparative Medicine at the Massachusetts Institute of Technology (MIT) and Department of Medical Oncology at the Dana Farber Cancer Institute and the Institutional Animal Care and Use Committee at both institutions.

<u>Data availability</u>
No large datasets were generated as part of this study. Any details regarding the data can be addressed directly to the corresponding author. The computer code below was used to aid IHC quantification. The values in bold were adjusted for the specific antibody used.

Antibody groovy

```
setImageType('BRIGHTFIELD_H_DAB');
//createSelectAllObject(true);
selectAnnotations();
//selectObjects { p -> p.getPathClass() == getPathClass("Tumor") && p.isAnnotation() };
setColorDeconvolutionStains('{"Name" : "H-DAB estimated", "Stain 1" : "Hematoxylin", "Values 1" : "0.655 0.668 0.353", "Stain 2" : "DAB", "Values 2" : "0.381 0.572 0.727", "Background" : " 242 242 242"}');
runPlugin('qupath.imagej.detect.cells.PositiveCellDetection', '{"detectionImageBrightfield": "Optical density sum",  "requestedPixelSizeMicrons": 0.5,  "backgroundRadiusMicrons": 10.0,  "medianRadiusMicrons": 0.0,  "sigmaMicrons": 1.5,  "minAreaMicrons": 10.0,  "maxAreaMicrons": 400.0,  "threshold": 0.1,  "maxBackground":
```

2.0, "watershedPostProcess": true, "excludeDAB": false, "cellExpansionMicrons": 5.0, "includeNuclei": true, "smoothBoundaries": true, "makeMeasurements": true, "thresholdCompartment": "Nucleus: DAB OD mean", "thresholdPositive1": 0.2, "thresholdPositive2": 0.4, "thresholdPositive3": 0.6000000000000001, "singleThreshold": true}');

Sex as a biological variable

An equal number of male and female mice were used for this study to ensure that the biological variable of sex did not confound the study.
